# Correlated evolution of distinct signals associated with increased social selection in female white‐shouldered fairywrens

**DOI:** 10.1002/ece3.8370

**Published:** 2021-11-23

**Authors:** John Anthony Jones, Karan J. Odom, Ian R. Hoppe, Doka Nason, Serena Ketaloya, Jordan Karubian

**Affiliations:** ^1^ Department of Ecology and Evolutionary Biology Tulane University New Orleans Louisiana USA; ^2^ Department of Neurobiology and Behavior Cornell Lab of Ornithology Cornell University Ithaca New York USA; ^3^ School of Natural Resources University of Nebraska Lincoln Nebraska USA; ^4^ Porotona Village Milne Bay Province Papua New Guinea; ^5^ Present address: Department of Psychology University of Maryland College Park Maryland USA

**Keywords:** fairywren, female ornamentation, female song, social selection

## Abstract

Conspicuous female signals have recently received substantial scientific attention, but it remains unclear if their evolution is the result of selection acting on females independently of males or if mutual selection facilitates female change. Species that express female, but not male, phenotypic variation among populations represents a useful opportunity to address this knowledge gap. White‐shouldered fairywrens (*Malurus alboscapulatus*) are tropical songbirds with a well‐resolved phylogeny where female, but not male, coloration varies allopatrically across subspecies. We explored how four distinct signaling modalities, each putatively associated with increased social selection, are expressed in two populations that vary in competitive pressure on females. Females in a derived subspecies (*M*. *a*. *moretoni*) have evolved more ornamented plumage and have shorter tails (a signal of social dominance) relative to an ancestral subspecies (*M*. *a*. *lorentzi*) with drab females. In response to simulated territorial intrusions broadcasting female song, both sexes of *M*. *a*. *moretoni* are more aggressive and more coordinated with their mates in both movement and vocalizations. Finally, *M*. *a*. *moretoni* songs are more complex than *M*. *a*. *lorentzi*, but song complexity does not vary between sexes in either population. These results suggest that correlated phenotypic shifts in coloration and tail morphology in females as well as song complexity and aggression in both sexes may have occurred in response to changes in the intensity of social selection pressures. This highlights increased competitive pressures in both sexes can facilitate the evolution of complex multimodal signals.

## Graphical abstract

Female white‐shouldered fairywrens of New Guinea have female‐specific plumage polymorphisms among allopatric populations. Combining a series of behavioral assays, song recordings, and long term banding data, we show that females of a derived subspecies in which females are ornamented (*M*. *a*. *moretoni*) are also more aggressive at the individual‐level, more coordinated at the pair‐level, sing more complex songs, and have shorter tails (a putative sexual signal) than the subspecies with the ancestral (unornamented) female phenotype (*M*. *a*. *lorentzi*).

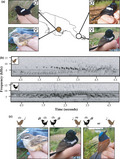

## 1 INTRODUCTION

Improving our knowledge of sexual dimorphism and phenotypic diversity depends on understanding signal evolution in both sexes. For this reason, better resolving the factors underlying variation in female signals in order to close the gap in the amount of research conducted between sexes is a core goal among contemporary evolutionary ecologists (Clutton‐Brock, 2007; Doutrelant et al., 2020; Odom et al., 2014; Tobias et al., 2012; Webb et al., 2016). One key question concerns the degree to which plumage‐based visual signals and vocal signals (e.g., song) are under direct selection in females versus being effectively dragged along by active selection on males (Darwin, 1871; Lande, 1980). While the nonadaptive hypothesis has received some support (Kraaijeveld, 2014; Poissant et al., 2010), there are cases in which female visual and vocal signals are different from or more elaborate than that of males (Brunton & Li, 2006; Heinsohn et al., 2005; Illes & Yunes‐Jimenez, 2012). Mounting evidence suggests that female ornamentation can evolve independently from that of males (Dale et al., 2015; Odom et al., 2014; Wilkins et al., 2020). Thus, exploring how selective pressures shared between sexes influences signal dynamics in similar or divergent ways is warranted to address different hypotheses explaining female signal evolution.

Animals often use multiple signal modalities simultaneously, either to reinforce a signal's message or to convey distinct messages to different receivers (Møller & Pomiankowski, 1993). Studies that concurrently evaluate multimodal signal evolution in the context of a known phylogeny are rare but are relevant to understand female trait evolution (e.g., Gomes et al., 2017, Hasegawa et al., 2017). For example, evidence from two or more populations with known phylogenetic history and different female signal states may provide insights into evolutionary relationships between female‐specific selection and signal expression, or selection that acts on females independently of that of males. Such selective pressures in females often takes the form of social selection, a process including competition for mates (i.e., sexual selection) as well as ecological resources (West‐Eberhard, 1979, 1983). Although social selection is increasingly used as a framework to evaluate the evolutionary significance of female ornamentation (Lyon & Montgomerie, 2012; Tobias et al., 2012), studies linking female signal evolution to changes in strength of social selection are rare (Doutrelant et al., 2020).

Among population shifts in the strength of the competitive environment may promote the correlated evolution of multiple, distinct signaling phenotypes in order to better cope with increased selection. Here, we define this correlated signal evolution as the emergence of a suite of signaling traits occurring in the same direction (e.g., evolution of more complex song along with more ornamented plumage), without implying that they emerge at the same time or via the same physiological mechanism. Rubenstein and Lovette (2009) used a qualitatively similar approach to show that female plumage ornamentation of different species of starling (Sturnidae) increases in parallel with intrasexual competition for mates. However, additional examples, particularly involving evolution of multiple signal types among different populations of a single species, are lacking.

**FIGURE 1:**
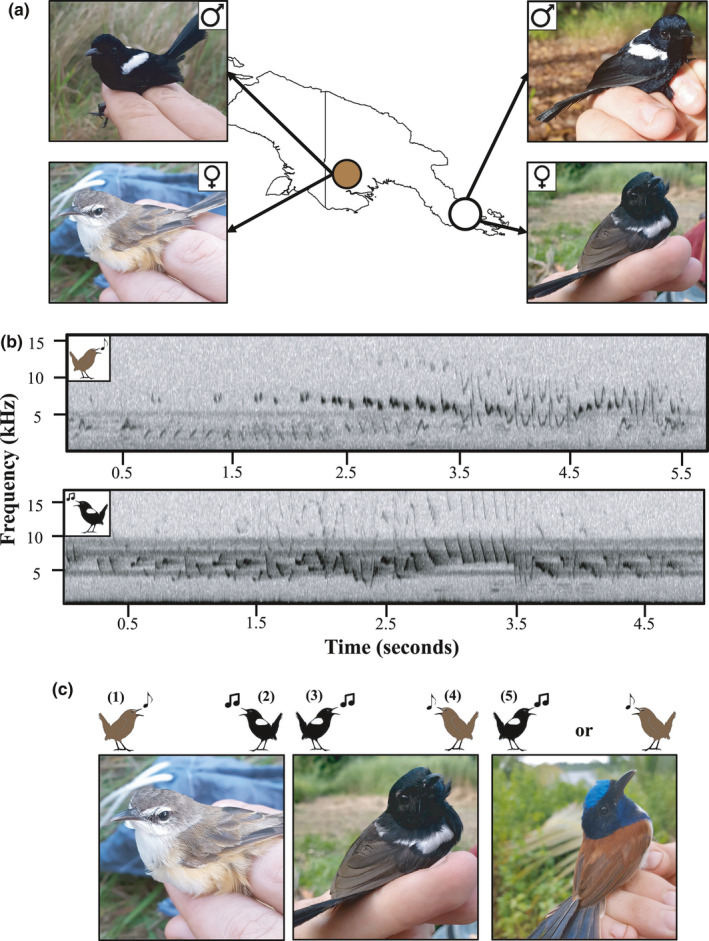
Visual representation of natural variation in signaling modalities between populations of white‐shouldered fairywren: (a) *Malurus alboscapulatus lorentzi* of Western Provence, Papua New Guinea (shaded circle; left) and *M*. *a*. *moretoni* of Milne Bay Province (open circle; right). Not depicted is a third female plumage phenotype/subspecies, that is, not involved in this study. (b) Representative example sonogram of *M*. *a*. *lorentzi* (top; brown cartoon) and *M*. *a*. *moretoni* (bottom, black cartoon) female song. (c) Experimental design of this study (illustrated by picture of each subspecies to represent the mount plumage presented and the cartoon fairywren to represent song exemplars as given in (b). Free flying fairywrens were presented with each treatment in randomized order: (1) *M*. *a*. *lorentzi* song and plumage mount, (2) *M*. *a*. *moretoni* song and *M*. *a*. *lorentzi* plumage, (3) *M*. *a*. *moretoni* song and plumage, (4) *M*. *a*. *lorentzi* song and *M*. *a*. *moretoni* mount, and (5) *M*. *cyanocephalus* plumage mount (to serve as a control) paired with the local phenotype's song phenotype

White‐shouldered fairywrens (*Malurus alboscapulatus*) of tropical New Guinea present a study system in which female signals vary between closely related populations and ancestral versus derived states of female plumage coloration can be inferred with confidence. This species is unusual, in that female plumage varies geographically, while male coloration remains unchanged (Enbody et al., 2019; Karubian, 2013; Rowley & Russell, 1997; Figure 1a). Female ornamentation in this species is derived: subspecies with ornamented females have evolved from unornamented female ancestors independent of any transition in male ornamentation (Driskell et al., 2011; Johnson et al., 2013; Karubian, 2013, E. D. Enbody *unpubl. data*). Throughout their range, both sexes sing and participate in territory defense (Rowley & Russell, 1997), but detailed analyses of song are lacking. In a subspecies in which females are ornamented alongside males (*M*. *a*. *moretoni*; Figure 1a), both sexes are more aggressive than a subspecies with unornamented females (*M*. *a*. *lorentzi*). Moreover, female, but not male (J. Boersma *unpubl. data*), testosterone concentrations are higher in the ornamented population (Enbody et al., 2018). Reverse sexual dimorphism on tail length is widespread in *Malurus*, highlighting the potential signaling function of shorter tails (Swaddle et al., 2000). For example, in the sister species to our focal fairywrens (red‐backed fairywrens; *M*. *melanocephalus*), shorter tails appear to be associated with social dominance among males (Karubian et al., 2009). Past work showed that unadjusted tail lengths of *M*. *a*. *moretoni* and *M*. *a*. *lorentzi* females did not vary statistically (Enbody et al., 2019) but did not account for body size differences. Finally, *M*. *a*. *lorentzi* commonly interacts with neighbors outside of the pair in a social context, whereas *M*. *a*. *moretoni* experiences limited extra‐territory interactions, and interactions that occur typically are agonistic (J. Boersma and J. A. Jones, *unpubl. data*). Together, these trends suggests that competitive pressures experienced by *M*. *a*. *moretoni* are greater than in *M*. *a*. *lorentzi* for both sexes, although the plumage change only occurs in one sex.

We combined a field‐based simulated territorial intrusion (STI) experiment, song analysis, and morphological measures to test the hypothesis that variation in social selection pressure differentially influences signal expression between sexes. Our overall prediction is that increased female plumage ornamentation is accompanied by parallel transitions in acoustic, behavioral, and morphological traits, consistent with increased social selection. We used STIs to explore the behavioral response of territorial residents to intruders of varying female plumage and song phenotype (Figure 1). We first predicted that *M*. *a*. *moretoni* would respond to STIs by both local and foreign phenotypes (1) more aggressively at the individual level (i.e., females and males independently will be more aggressive in this subspecies) and (2) with greater pair coordination than would *M*. *a*. *lorentzi*, consistent with Enbody et al. (2018). If these traits evolve in concert, we also predicted increased song complexity, but shorter body‐size adjusted tail length in female *M*. *a*. *moretoni*, in tandem with greater plumage ornamentation. Finally, if our initial predictions are supported, we predict that the combination of the *M*. *a*. *moretoni* plumage and song will elicit the strongest aggressive response in both populations. Support for these predictions would suggest that increases in signal complexity and female ornamentation are associated with an increase in joint territory defense. Moreover, this would suggest that these distinct signaling modalities have undergone correlated evolutionary transitions in a direction consistent with increased social selection.

## 2 METHODS

We studied two populations of white‐shouldered fairywrens from May to July 2018: *M*. *a*. *moretoni* (in which both males and females are ornamented) in Garuahi Village, Milne Bay Province (Lat: −10.2187, Long: 150.4843) and *M*. *a*. *lorentzi* (in which females are unornamented; Figure 1); in Obo Village, Western Province, Papua New Guinea (−7.6047, 141.3090). We captured fairywrens via mist‐nets, banded each bird with a unique combination of colored leg bands for individual identification, and measured tail and tarsus lengths (±0.01 mm).

### 2.1 Behavioral assay protocol

Enbody et al. (2018) previously used a pair‐level STI with paired male and female mounts of both ornamented and unornamented phenotypes. In both cases, the mounts were coupled with audio duet (male and female) exemplars of the resident (local) song and were not presented with the other subspecies’ song. The authors found that mount phenotype (i.e., ornamented versus unornamented) did not affect how pairs responded. We built upon this earlier study by assessing behavioral responses to varying female plumage and song phenotype stimuli, without any male stimuli. This distinction is noteworthy because, while an intruding male and female pair may represent an equal threat to both sexes, a lone female could be perceived as more of a threat to resident females than resident males (e.g., Mennill, 2006). Thus, one biological interpretation of our design is that females perceive the simulated intruder as a possible usurper (e.g., Guo et al., 2020). However, as our treatments were a combination of female plumage and song exemplars only (i.e., no male stimuli), expectations for how the mated male may respond to these intrusions is less straightforward. Although it is possible males may perceive the simulated intruder as a possible reproductive opportunity, it is also possible that males would be as aggressive as their partner to strength existing pair bonds (Ens et al., 1996). Moreover, equivalent levels of aggression aimed towards either sex are a general strategy males employ to ensure control of the territory, thus maintaining partnerships (Guo et al., 2020; Hau et al., 2004).

Our experimental design follows that of Greig et al. (2015), who compared male behavior and ornamentation in two allopatric subspecies of red‐backed fairywrens (*M*. *melanocephalus*). Free‐flying pairs were repeatedly assayed (without replacement) with one of with five possible treatments, comprised of female plumage and song combinations in randomized order with ~3 days (range: 2–5 days) between trials (Figure 1c): (1) *M*. *a*. *lorentzi* song and plumage mount, (2) *M*. *a*. *moretoni* song and *M*. *a*. *lorentzi* plumage, (3) *M*. *a*. *moretoni* song and plumage, (4) *M*. *a*. *lorentzi* song and *M*. *a*. *moretoni* mount. Additionally, we presented (5) an emperor fairywren (*M*. *cyanocephalus*) female mount paired with local (with respect to population) white‐shouldered fairywren song to serve as a heterospecific plumage control. These treatments were coded as a playback scenario that simulated the local versus foreign (relative to the focal population) subspecies plumage and song for analysis. We did not use an acoustic control, as neither subspecies responds to heterospecific playback. Our aim was to present pairs with each of the five treatments; we did not include assay trials if one member of the mated pair did not respond, regardless of sex.

We broadcasted song exemplars previously recorded (during other field seasons; no playback exemplars were recorded from individuals within this study from this year) and broadcast from the mount location using an Ultimate Ears Roll 2 speaker (Irvine, CA, USA). Songs were randomly chosen, after excluding song from the focal individual and neighboring territories. Apart from the emperor fairywren phenotype, mount exemplars (*n* = 4 of each phenotype; 12 total) were the same three‐dimensional painted bird models used in Enbody et al. (2018).

Free‐living pairs were detected without audio duet playback in most cases (exposing birds to duets prior to behavioral assays did not significantly influence the response (*p* > .30), after which mounts were placed ~1.5 m off the ground and we retreated ~25 m (range: 20–40 m) and minimized exposure. For ~5 min of playback and 5 min of silence (post playback), we recorded latency to respond and approach (song response is not discernible without visual observation), proportion of time within 5 m of the mount, and the average distance to mount (calculated as the average time spent within each categorical distance class: <0.5 m, 0.5–5 m, 5–10 m, 10–15 m, and >15 m) separately for the free‐flying male and female. Additionally, we noted individual flybys within 2 m of the mount as well as songs sung with and without the mate. To quantify a pair's degree of coordination in response to treatment, we noted the latency between the male and female responses (i.e., shorter latency is associated with more coordinated behaviors) as well as the proportion of time the pair spent together (within 1 m of each other) throughout the trial. We recorded other pair coordination behaviors, including allopreening, duetting, and leapfrogs (where one bird jumps over another on the same perch) as rates (events per min). We noted whether a male attempted to court the mount (i.e., visual displays: puff‐shoulder display, display flights, and petal carries), but found there was no relationship among treatments nor between subspecies and display rate (all *p* > .32), and thus exclude these results from this study.

### 2.2 Song analysis

Female songs were recorded using a Marantz PMD 661 Mk II (96kHz sampling rate, 24‐bit depth; D&M Professional) with a Sennheiser ME66 shotgun microphone and K6 power module (Sennheiser Electronic Corporation). For each population, we created playback exemplars from five individual females recorded in 2017. Each exemplar consisted of a single female song repeated at 10 s intervals for ~5 min total (exact time varied; trial length was accounted for to calculate behavior rates). We used Audacity to filter out noise below 500 Hz and standardize amplitude.

We calculated element diversity and several other metrics representing song complexity to compare overall song structure of male and female songs recorded in 2018 (both subspecies) and 2019 (*M*. *a*. *moretoni* only). We first standardized to a sample rate of 44.1 kHz and bit depth of 16 and then used Raven Sound Analysis Software v1.6 (Center for Conservation Bioacoustics, 2019) to select every element in each song. We defined an element as a single, continuous trace on a spectrogram separated from other elements by a visible break in time. Measurements in Raven were made using a Hanning window with a 512 FFT and 90% overlap for a time resolution of 1.161 ms, and a frequency resolution of 86.1 Hz. From these selections, we extracted robust (energy‐based) time and frequency measurements of each element in Raven. We then transferred the selections into R using the *Rraven* package (Araya‐Salas, 2017), where we extracted additional acoustic parameters via the *warbleR* package (Araya‐Salas & Smith‐Vidaurre, 2017). We removed highly correlated acoustic variables (*r* ≥ |0.95|), resulting in 24 acoustic variables extracted for each element, including measures of frequency, frequency bandwidth, frequency modulation, time, duration, and entropy (Table S1). These element‐level metrics were used to estimate element diversity from a 2‐D acoustic trait space (Keen et al., 2021). We also used the song_param function in *warbleR* to extract song‐level metrics, resulting in the following final set of metrics calculated for the entirety of each song: (1) song duration, (2) mean element duration, (3) mean peak frequency, (4) frequency range (calculated as the difference between the highest 95% (i.e., maximum frequency) and the lowest 5% (i.e., minimum) frequency values of all elements in each song), and (5) element diversity. These five song‐level metrics were used in subsequent statistical analyses to compare subspecies differences in song structure.

To calculate element diversity, we created an element‐level acoustic space via an unsupervised random forest that included every element‐level acoustic parameter in R package *randomForest* (Liaw & Wiener, 2002). The random forest analysis was run with the following specifications: 10,000 trees, minimal node size of 1, Gini impurity index as split rule, five randomly sampled variables at each split and out‐of‐bag proximity. This process created a proximity matrix that was transformed into a set of five vectors using classic multidimensional scaling (MDS) via the cmdscale function in the *stats* R package. We used the MDS vector to create a 2‐D acoustic space containing all elements of all songs. The area that the element encompasses is indicative of its diversity, with larger areas being more diverse. We extracted 95% minimum convex polygon of the areas defined by the elements for each song using the function mcp in the R package *adehabitatHR* (Calenge, 2015). This method was ground‐truthed using datasets of known element diversity by Keen et al. (2021).

### 2.3 Statistical analysis

We reanalyzed a subset of morphological descriptive data available from Enbody et al. (2019) to test whether tail length varies between subspecies while controlling for body size and sex. We controlled for body size by regressing tail length against tarsus length and performed an independent samples t‐test to test for sex‐specific differences in residual tail length between subspecies.

We explored response to simulated intruders at the level of the individual (i.e., male or female response independent of one another) and pair (i.e., the joint, coordinated response) separately using two principal components analyses (PCA). In the Individual‐PC, we included the following individual‐level metrics: latency to respond, flyby rate, solo and duet song rate, time within 5 m of the mount, and average distance from the mount. For the Pair‐PC, we included the following pair‐level metrics: the difference in time between male and female response to the mount (denoted as latency lag), the proportion of time spent together versus apart during the trial, and the rates of allopreening, leapfrogging, and duetting. Duets were included in both PCs to account for the fact that some individuals may have intended to sing individually but were subsequently joined in song by the partner. For all behavioral PCs, we normalized each response variable by log+1 transformation followed by centering and scaling prior to running the PCA (Filardi & Smith, 2008; Uy et al., 2009).

We first assessed the effects of stimulus treatment type, subspecies, and sex of the responding bird on the top principal components using linear mixed effects models using the *lme4* package in R (Bates et al., 2015; R Core Team, 2020). Individual ID (individual analysis only) was nested within pair ID as random intercepts in our models, as well as mount ID (12 available mount phenotypes, 4 per treatment) and female song stimulus ID (10 available song recordings, 5 per subspecies) to reduce the effect of pseudoreplication (see Kroodsma et al., 2001). Covariates including pair breeding stage (i.e., breeding versus nonbreeding), time of day of the behavior assay, the order in which each treatment was given to a pair, and whether or not another male/juvenile approached during the assay did not significantly predict the behavioral response, and thus were not included in the final model. Residuals of the full model did not violate assumptions of normality nor homoscedasticity. The significance of each model was evaluated using a χ^2^ test in the *car* package in R (Fox & Weisberg, 2011).

To explore whether song elaboration varies between sexes and subspecies, we created a reduced set of song‐level acoustic variables via a PCA on a correlation matrix of our acoustic variables and extracted the first three principal components for further analyses. We then ran three separate linear mixed effects models for each principal component, with those components as dependent variables and with sex and subspecies as fixed effects. We included individual ID, population, and year recorded as random intercepts in each model. We additionally tested for a significant interaction between subspecies and sex; upon finding, the interaction was not statistically significant, we ran our models without the interaction term.

### 2.4 Ethics statement

Our study was carried out in strict accordance with the guidelines established by the Tulane University Institutional Animal Care and Use Committee (#0395R2). We minimized handling time for each individual in order to reduce physical stress and harm.

## 3 RESULTS

### 3.1 Morphological comparison

Female *M*. *a*. *moretoni* tails are shorter on average than those of *M*. *a*. *lorentzi* when accounting for body size (*t* = 5.32, df = 199, *p* < .001), whereas male body‐size adjusted tail length does not significantly vary between populations (*t* = 0.11, df = 206, *p* = .91).

### 3.2 Individual response to STI

We excluded trials when a bird did not respond to the stimulus, resulting in 91 *M*. *a*. *lorentzi* and 102 *M*. *a*. *moretoni* female responses and 97 *M*. *a*. *lorentzi* and 102 *M*. *a*. *moretoni* male responses. The first three PCs explained 76.9% of the variation in individual responses (Table 1). Higher scores for Individual‐PC1 are associated with individuals that are more aggressive, characterized predominately by a faster response to the stimulus, aggressive posturing (i.e., birds spending more time close to the mount), and more frequent flybys. Higher scores for Individual‐PC2 indicate individuals that sing more solo songs and tend to fly by the mount more often. Individual‐PC3 appears to be associated with individuals that favor threats rather than direct aggression, such that higher scores correspond to quicker response times and greater singing rates, but with a tendency to remain >5 m from the mount.

**TABLE 1:** Loading scores for the principal component analysis exploring (1) individual and (2) paired response to STIs

	Individual‐PC1	Individual‐PC2	Individual‐PC3	Pair‐PC1	Pair‐PC2
Eigenvectors (SD)	1.594	1.045	0.992	1.413	0.999
Proportion of Variance	0.423	0.182	0.164	0.399	0.200
Latency	−0.381	0.086	−0.555		
Flyby (min^−1^)	0.364	0.309	−0.132		
Time within 5 m of mount	0.553	0.077	−0.327		
Avg. distance from mount	−0.580	−0.042	0.225		
Solo songs (min^−1^)	0.010	0.771	0.515		
Duet (min^−1^)	0.283	−0.544	0.503	0.591	0.118
Latency Lag				−0.340	0.680
Allopreen (min^−1^)				0.466	0.440
Leapfrog (min^−1^)				0.395	0.354
Proportion of time together			0.404	−0.452	

Note

Individual‐PC: Latency: delay in response to playback stimuli. Flyby: the rate at which individuals flew past 2 m of the mount. Pair‐PC: Latency lag: difference in time between the first responses to playback stimulus by each member of the focal pair; proportion of time: time spent physically together and behaving in a coordinated fashion versus time spent physically apart. Note: duets are a component of both PCs.

**FIGURE 2:**
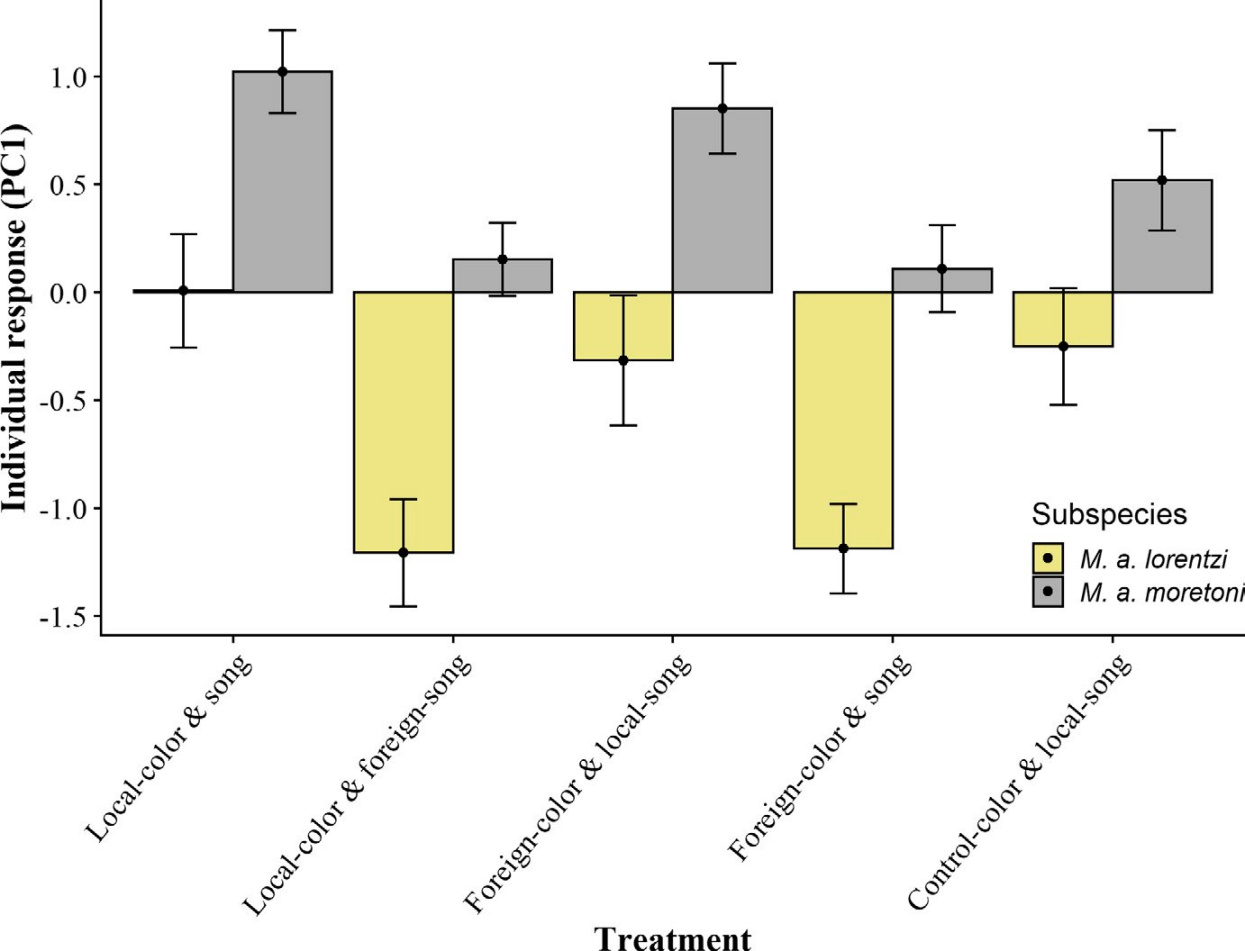
White‐shouldered fairywren behavioral response (PC1) to STIs with respect to treatment and subspecies. Bars represent mean ± standard error

There were no significant interactions among treatment, sex, or subspecies, and thus we excluded these interaction terms from the final models. The lack of interaction between treatment and subspecies suggests that the two subspecies respond to treatments in a similar fashion. Thus, each subspecies’ response is determined by whether the stimulus treatment is local or foreign, rather than presented phenotype was ornamented or not. Treatment predicted Individual‐PC1 and Individual‐PC3 (Table 2); individuals of both sexes are more aggressive when exposed to treatments that contain a local song (with respect to population; all *p* < .01, Figure 2a, Table S2). Additionally, *M*. *a*. *moretoni* respond overall more aggressively than *M*. *a*. *lorentzi* (higher PC1 scores; Figure 2b). Individual‐PC3 varies between sexes, such that males are overall more responsive to STIs on this axis. Finally, Individual‐PC2 is not predicted by treatment or subspecies, but we found a near significant trend that females tend to have lower PC2 scores, indicative of fewer flybys of the exemplar coupled with increased duetting (rather than individual solo songs) when responding to playback (*p* = .06, Table 2).

**TABLE 2:** Summary statistics detailing the effects of treatment, subspecies, and sex on the behavioral response of individuals at both the individual (top) and pair (bottom) levels

	Fixed effects	Sum Sq	Mean Sq	*F*	df	*p*
Individual response to STI						
Individual‐PC1	Treatment	74.573	18.643	20.958	4, 41.485	**<.001**
	Subspecies	9.006	9.006	10.124	1, 41.527	.**003**
	Sex	0.311	0.311	0.35	1, 39.795	.555
	*Random effects:*	*Variance*				
	Bird ID/Pair ID	<0.001				
	Pair ID	1.16				
Individual‐PC2	Treatment	4.474	1.119	1.555	4, 37.247	.207
	Subspecies	0.594	0.594	0.825	1, 39.644	.369
	Sex	2.754	2.754	3.828	1, 38.079	.*058*
	*Random effects:*	*Variance*				
	Bird ID/Pair ID	0.09				
	Pair ID	0.21				
Individual‐PC3	Treatment	9.207	2.302	4.199	4, 39.245	.**006**
	Subspecies	0.476	0.476	0.869	1, 38.272	.357
	Sex	3.784	3.784	6.903	1, 39.912	.**012**
	*Random effects:*	*Variance*				
	Bird ID/Pair ID	0.01				
	Pair ID	0.34				
Pair response to STI						
Pair‐PC1	Treatment	24.418	6.105	5.595	4,45.177	.**001**
	Subspecies	2.163	2.163	1.983	1,41.274	.167
	*Random effects*	*Variance*				
	Pair ID	0.74				
Pair‐PC2	Treatment	3.74	0.934	1.269	4,47.859	.375
	Subspecies	6.62	6.621	8.996	1,39.846	.**001**
	*Random effects*	*Variance*				
	Pair ID	0.08				

### 3.3 Pair response to STI

We excluded pair responses in which only one member of the pair responded to the stimulus, resulting in 90 trials completed in *M*. *a*. *lorentzi* and 102 in *M*. *a*. *moretoni*. For these trials, the first two PCs explain ~60% of the variation in pair responses to STI (Table 1). Higher scores for Pair‐PC1 indicate a greater degree of pair coordination. This was characterized by more frequent duets and allopreening and a greater proportion of time spent together. Pair‐PC2 appears to be associated with birds that spend a greater proportion of time together with a smaller latency lag (i.e., a more synchronous response to the stimulus), but also perform allopreening and leapfrog behaviors less frequently. We interpret higher Pair‐PC2 scores as indicating a more coordinated and aggressive response.

**FIGURE 3:**
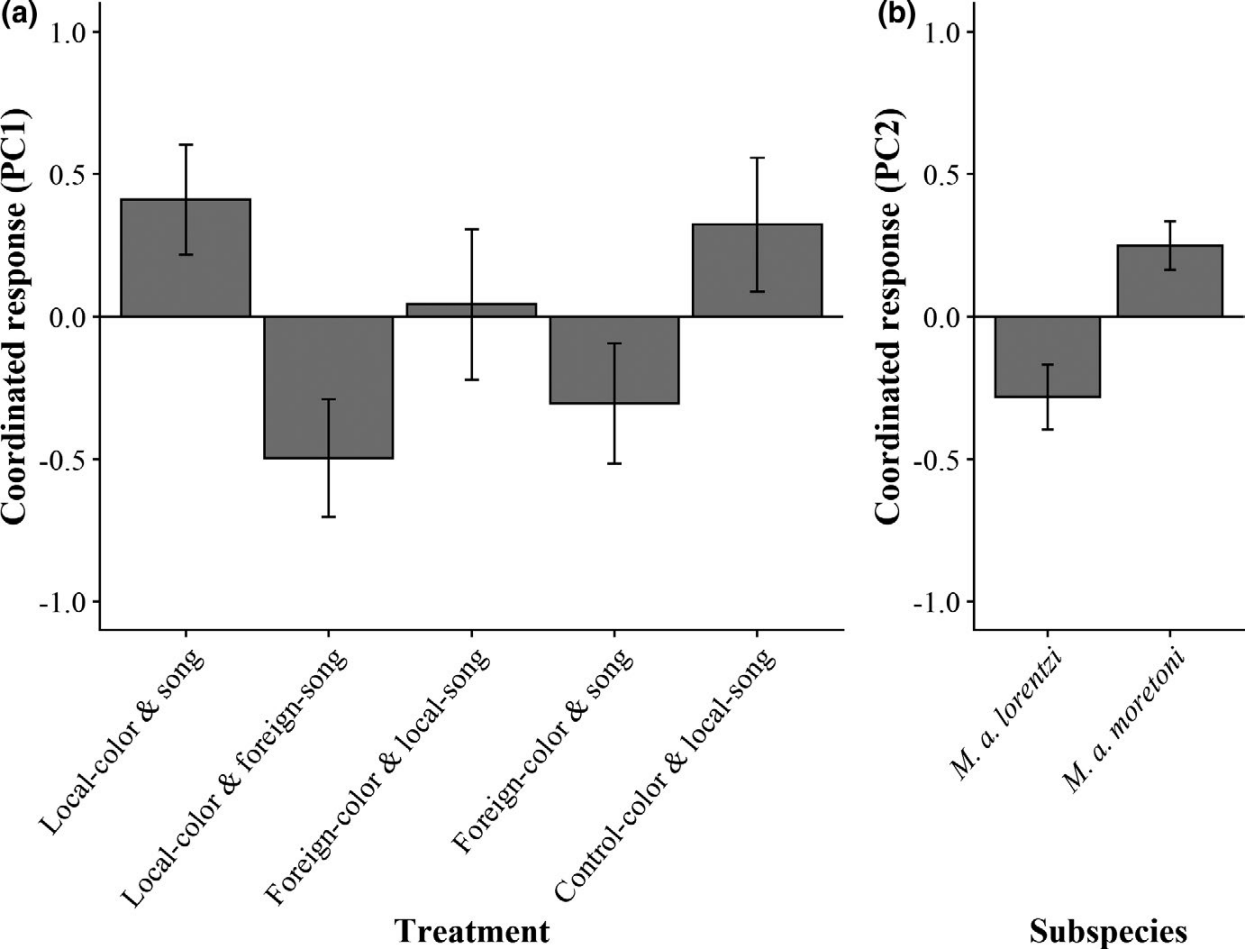
White‐shouldered fairywren pair coordinated response to STIs with respect to (a) treatment for Pair‐PC1 and (b) subspecies for Pair‐PC2. Bars represent mean ± standard error

We found no significant interaction between treatment and subspecies and did not include these interaction terms in the final model. Treatment predicted Pair‐PC1, but not Pair‐PC2 (Table 2). Similar to Individual‐PC1, the differences among treatments are driven by the presence of the local phenotype's song: pair coordination is higher in STIs when a local song (with respect to the focal population) is broadcasted, regardless of color mount presented (all *p* < .01, Figure 3a, Table S2). Subspecies, but not treatment, predicted Pair‐PC2; *M*. *a*. *moretoni* are more coordinated in response to simulated intrusions than are *M*. *a*. *lorentzi* pairs (Figure 3b, Table 2).

### 3.4 Song variation

We recorded 45 female songs (*n* = 11 individuals) and 26 male songs (*n* = 14) in one population of *M*. *a*. *lorentzi* and 36 female (9) and 32 male songs (11) between two populations of *M*. *a*. *moretoni*. We present the top three principal components (Table 3), explaining 69.7% of the total acoustic variation. The first component explains 34.2% of the total variation and captures element structure and variation. High scores for this component indicate songs with longer and more diverse elements separated from one another by comparatively short periods of silence. Highly scoring songs also show a greater frequency range at the element level, although the mean peak frequency of the entire song is lower. Song‐PC2 is driven predominately by the frequency range at the song level, with higher scores corresponding to songs with a greater frequency range. Finally, negative Song‐PC3 scores are associated with longer songs, while positive scores tend to correspond to songs with longer elements with greater frequency ranges despite these not loading as highly as duration.

**TABLE 3:** PCA eigenvalues, proportion of variation explained, and loading factors for the top three components of acoustic variation

Acoustic parameters	Song‐PC1	Song‐PC2	Song‐PC3
Eigenvalues	1.547	1.202	1.020
Proportion of variance	0.342	0.206	0.148
Song duration	0.287	0.297	−0.576
Mean element duration	0.451	0.056	0.472
Mean peak frequency	−0.414	0.393	0.389
Frequency range (song‐level)	−0.156	0.682	−0.083
Mean frequency range (element‐level)	0.425	0.281	0.479
Gap duration	−0.436	0.249	0.025
Element diversity	0.384	0.385	−0.240

Note

Song and element duration are defined as the units of time for the length of the song and mean element length. Frequency range at the song and element levels are calculated from the highest 95% and the lowest 5% frequency value for all elements in each song. Gap duration is the mean unit of time separating elements within a song. Element diversity is calculated as a 95% minimum complex polygon surrounding song structure at the element‐level acoustic space.

We found no sex differences across the models but did find a significant difference between subspecies in Song‐PC1 and ‐PC3 (Table 4 **)**. Both Song‐PCs are higher in *M*. *a*. *moretoni*: their songs contain longer, more diverse, and more complex elements than those sung by *M*. *a*. *lorentzi*, whose songs are longer and occur at a greater peak frequency on average than their ornamented counterparts.

**TABLE 4:** Model estimates for how acoustic parameters vary between sex and subspecies

	Estimate	SE	*t*‐value	*F*	df	*p*
Song‐PC1						
Sex	−1.034	0.264	4.124	0.281	1, 49.35	.598
Subspecies	0.127	0.24	49.350	36.69	1, 3.772	.**005**
Song‐PC2						
Sex	0.056	0.221	39.849	0.576	1, 49.505	.452
Subspecies	−0.206	0.272	49.505	0.237	1, 45.484	.628
Song‐PC3						
Sex	0.149	0.173	38.918	1.346	1, 48.943	.252
Subspecies	0.244	0.211	48.943	6.31	1, 44.32	.**016**

## 4 DISCUSSION

Many female passerines exhibit elaborate plumage and sing complex songs, but only recently have we begun to rigorously explore the evolutionary antecedents of these traits. We investigated four distinct phenotypic traits in two subspecies of *M*. *alboscapulatus*, testing whether those traits diverged in the same direction (i.e., increasing in ornamentation and complexity) in association with an increase in social selective pressure. Each of the four signals we investigated (tail length, song complexity, aggression, and pair coordination) are putatively involved in social interactions. In females, all four traits exhibit a correlated evolutionary transition that accompanies an increase in plumage ornamentation from a drab ancestral state to a derived, ornamented state. For males, however, neither coloration (Enbody et al., 2018) nor tail length (the current study) differs between populations, although males of the population with ornamented females (*M*. *a*. *moretoni*) are more aggressive and sing more complex songs than male *M*. *a*. *lorentzi*. Finally, we were unable to detect a difference between males and females in both aggression and song complexity within each respective population. Taken together, these results suggest that both sexes experience increased competitive pressures. However, the subspecies differences in morphology found only in females may infer they have a greater need to “catch up” with the phenotypic signals that males display. In other words, the intensity of selection may be similarly strong in both sexes, consequently driving increased territorial aggression and song complexity alongside an increase in female plumage ornamentation and a decrease in overall tail length (sensu Balmford et al., 2000; Karubian et al., 2009). Moreover, these results suggest that the most parsimonious explanation for this finding is that increased competitive pressures are experienced by female fairywrens is promoting several, potentially reinforcing signal types: female divergence in four phenotypic traits commonly associated with social selection, either via sexual selection (i.e., intrasexual competition for mates, intersexual mate choice) or competition (e.g., over ecological, nonreproductive resources), is unlikely to be happenstance. However, at this time we are unable to determine the order in which these traits arose evolutionarily, nor whether there is a common physiological mechanism (e.g., testosterone) underlying these transitions.

Discerning the explicit degree of competition that occurs within each population and how it may or may not vary between sexes represents an important goal for future work in this system. Nonetheless, these results provide an opportunity to better understand how differences in apparent social selection pressure at the population or subspecies level may be associated with female phenotypic divergence. Several key differences in the life history of our two study populations likely contribute to differing social selection pressures on females. First, the population with dull female plumage (*M*. *a*. *lorentzi*) experiences pronounced seasonal (monsoonal) variation whereas *M*. *a*. *moretoni* occurs in a more stable climate (Enbody et al., 2019) which in turn is generally associated with year‐round territoriality, reduced sexual promiscuity, and more equal effort between the sexes (Stutchbury & Morton, 2001; Tobias et al., 2016). Moreover, female *M*. *a*. *lorentzi* exhibit lower concentrations of circulating testosterone relative to *M*. *a*. *moretoni* (Enbody et al., 2018), consistent with a lower degree of intrasexual selection pressure.

Increases in social cohesion within mated pairs often arise in the context of greater competitive pressures for ecological resources (Griffith, 2019). *M*. *a*. *moretoni* songs consist of longer, more diverse elements than those of *M*. *a*. *lorentzi*, which may be perceived as higher quality (e.gBallentine, 2006; Searcy & Nowicki, 2006). Although both of our focal fairywren populations duet, we found evidence for greater coordination among pairs in *M*. *a*. *moretoni*. The occurrence of duetting is strongly associated with year‐round territoriality and strong social bonds, and playback studies support that duets in many species are used in territory defense and maintaining a functioning partnership (Dahlin & Benedict, 2014; Tobias et al., 2016; Wachtmeister, 2001). Although alternative explanations for acoustic differences between populations exist, we believe these are unlikely the key determinant of phenotypic divergence observed here. For example, while aspects of the ecological environment may influence the efficacy of acoustic signals (Endler, 1993), both subspecies sing predominately in open grasslands where transmission efficacy is similar (J. A. Jones *pers. obs*.).

In contrast to our predictions, local vocal signals elicited a uniformly stronger response than did plumage coloration, suggesting a prioritization of acoustic over visual signals in this system. Both subspecies recognized and responded aggressively to the song phenotype of the opposite population, suggesting elements of the acoustic structure are retained between populations and are used in species recognition contexts. Like red‐backed fairywren males (Greig et al., 2015), our focal species discriminated between song types, but not color, at the subspecies level. Several nonmutually exclusive explanations for this result are possible. The capacity for song to elicit aggressive behaviors may be strong enough to swamp other types of signals, such that variation in those signals is functionally irrelevant in aggressive contexts involving song. Alternatively, individuals may gauge the quality of the territory intruder and appropriately respond based on their own coloration. For example, male red‐backed fairywrens are less aggressive towards potential rivals that have lower‐quality plumage (Karubian et al., 2008). Of course, female plumage may be assessed by potential mates rather than by rivals (e.g., Thys et al., 2020). These results are not an indication that visual signals are irrelevant in female competitive contexts, but they do suggest that signaling function of female *M*. *a*. *moretoni* plumage ornaments warrants further study.

Selection should favor a multidimensional integrated phenotype that better equips individuals to respond to changing selective pressures. Phenotypic integration occurs when multiple life‐history characteristics, including morphological, physiological, and/or behavioral traits covary in a manner, that is, more adaptive than if the traits were decoupled (see reviews in McGlothlin & Ketterson, 2008; Pigliucci & Preston, 2004). Testosterone is a possible candidate hormone that plays a role in facilitating phenotypic integration (reviewed in Lipshutz et al., 2019). Higher concentrations of testosterone in *M*. *a*. *moretoni* females compared to *M*. *a*. *lorentzi* females is consistent with a role in coordinating expression of both plumage and behavior (Enbody et al., 2018). Moreover, Boersma et al. (2020) were able to experimentally induce the white shoulder ornament in the population with unornamented females (*M*. *a*. *lorentzi*) via an exogenous testosterone implant. However, ornament expression in unornamented females is not a naturally occurring phenotype, and it remains unclear if androgens have an influence on ornament expression in populations with naturally occurring female ornamentation. It is additionally unclear what role testosterone plays on tail length or acoustic performance (e.g., Dittrich et al., 2014 and references therein). Thus, exploring the physiological underpinnings of multimodal signal expression remains an exciting avenue for future work in this system to determine if these signals evolve independently of each other or if there is a common physiological mechanism.

More broadly, although we often characterize female signals by the degree to which they resemble those of males, in many instances selection may often operate on both sexes independently (Cain & Rosvall, 2014; Dale et al., 2015; Rosvall, 2011). As such, a more nuanced focus on how female‐specific selection occurs and directly influences the evolution of female signals per se is appropriate. This study, which highlights this evidence for direct selection occurring on females by detailing how multiple signaling phenotypes exhibit correlated evolutionary transitions in the context of increasing social selection pressures, represents one step in this direction.

## CONFLICT OF INTEREST

The authors have no conflicts of interests to disclose.

## AUTHOR CONTRIBUTIONS

**John Anthony Jones:** Conceptualization (lead); Data curation (lead); Formal analysis (lead); Funding acquisition (supporting); Investigation (lead); Methodology (lead); Project administration (lead); Writing‐original draft (lead); Writing‐review & editing (lead). **Karan J. Odom:** Software (lead); Supervision (supporting); Validation (supporting); Visualization (lead); Writing‐review & editing (equal). **Ian R. Hoppe:** Formal analysis (supporting); Investigation (supporting); Methodology (supporting); Writing‐review & editing (equal). **Doka Nason:** Methodology (supporting); Project administration (supporting); Resources (equal). **Serena Ketaloya:** Methodology (supporting); Project administration (supporting); Resources (equal). **Jordan Karubian:** Conceptualization (supporting); Funding acquisition (lead); Methodology (supporting); Project administration (supporting); Resources (supporting); Writing‐review & editing (equal).

## Supporting information

Appendix S1

## Data Availability

Data and R script files are available publicly Dryad at https://doi.org/10.5061/dryad.hdr7sqvhb.
