# Supplementary material for: Correlated evolution of distinct signals associated with increased social selection in female white‐shouldered fairywrens
Source: Ecol Evol. 2021 Nov 23;11(23):17352–63. doi: 10.1002/ece3.8370 (PMC8668759; doi:10.1002/ece3.8370)
Supplement: Supplementary file 1 — Appendix S1 [file ECE3-11-17352-s001.docx]

# **Supplemental material**

Table S1. Metrics extracted for each element in white-shouldered fairywren (*Malurus alboscapulatus*) songs. Definitions are based on the software packages from which the acoustic metrics were extracted: Raven Sound Analysis Software v1.6 (Charif et al. 2003; Center for Conservation Bioacoustics 2019) and the warbleR package in R (Araya-Salas and Smith-Vidaurre 2017).

| **Metric** | **Program from** | **Definition** |
| --- | --- | --- |
|  |  |  |
| Bandwidth 90% (Hz) | Raven | The difference between the 5% and 95% frequencies (Hz), where 5% and 95% frequency are the frequencies that divide the selection into two frequency intervals containing 5% and 95% and 95% and 5% of the energy in the selection (Hz), respectively, and therefore represent minimum and maximum frequencies. Therefore, Bandwidth 90% is an energy-based estimate of frequency bandwidth. |
| Maximum frequency (Hz) | Raven | The frequency at which Maximum Power occurs within the selection. If Maximum Power occurs at more than one time and/or frequency, the lowest frequency at Maximum Time at which Maximum Power occurs (Hz). |
| Number of inflection points | Raven | Number of times the slope changes sign in the Peak Frequency Contour Slope series of numbers. |
| Aggregate entropy (bits) | Raven | Entropy calculated using the total energy in a frequency bin over the full time span of the signal, calculated using the formula: S = PSD(f,t)/sum_over_f(PSD(f,t)) * log2( PSD(f,t)/sum_over_f(PSD(f,t))). |
| Average entropy (bits) | Raven | The average entropy calculated for all spectrogram slices within the selection bounds, where entropy is computed on a per-spectrogram-slice basis, using the formula: S = PSD(f,t)/sum_over_f(PSD(f,t)) * log2( PSD(f,t)/sum_over_f(PSD(f,t))). |
| Maximum entropy (bits) | Raven | The maximum entropy calculated for a spectrogram slice within the selection bounds, where entropy is computed on a per-spectrogram-slice basis, using the formula: S = PSD(f,t)/sum_over_f(PSD(f,t)) * log2( PSD(f,t)/sum_over_f(PSD(f,t))). |
| Minimum entropy (bits) | Raven | The minimum entropy calculated for a spectrogram slice within the selection bounds, where entropy is computed on a per-spectrogram-slice basis, using the formula: S = PSD(f,t)/sum_over_f(PSD(f,t)) * log2( PSD(f,t)/sum_over_f(PSD(f,t))). |
| 1st quartile time | warbleR | The time at which the signal is divided in two time intervals of 25% and 75% energy respectively (in s). |
| Time interquartile range | warbleR | Time range between 'time.Q25' and 'time.Q75' (in s), where time.Q25 and time.Q75 are the times within the selection at which the signal is divided in two time intervals of 25% and 75% or 75% and 25% energy respectively, and therefore represents an energy-based estimation of duration. |
| Median frequency | warbleR | The frequency at which the signal is divided in two frequency intervals of equal energy (in kHz) |
| Interquartile frequency range | warbleR | Frequency range between 'freq.Q25' and 'freq.Q75' (in kHz), where freq.Q25 and freq.Q75 are the frequencies at which the signal is divided in two frequency intervals of 25% and 75% and 75% and 25%energy respectively (in kHz), and there represents an energy-based estimate of frequency range. |
| Dominant frequency range | warbleR | Range of dominant frequency measured across the acoustic signal |
| skewness | warbleR | Asymmetry of the spectrum (see note in specprop R package description) |
| time entropy | warbleR | Energy distribution on the time envelope. Pure tone ~ 0; noisy ~ 1. |
| Minimum of the dominant frequency | warbleR | Minimum of dominant frequency measured across the acoustic signal |
| standard deviation of frequency | warbleR | Standard deviation of the frequency weighted by amplitude (in kHz). |
| Kurtosis | warbleR | Peakedness of the spectrum |
| Spectral entropy | warbleR | Spectral entropy calculated as the product of time and spectral entropy where spectral entropy = Energy distribution of the frequency spectrum. Pure tone ~ 0; noisy ~ 1. |
| Spectral entropy | warbleR | Spectral entropy calculated as the energy distribution of the frequency spectrum. Pure tone ~ 0; noisy ~ 1. |
| spectral flatness | warbleR | Similar to spectral entropy (energy distribution of the frequency spectrum). Pure tone ~ 0; noisy ~ 1. |
| modulation index | warbleR | Calculated as the cumulative absolute difference between adjacent measurements of dominant frequencies divided by the dominant frequency range. 1 means the signal is not modulated. |
| Start dominant frequency |  | Dominant frequency measurement at the start of the signal (kHz). |
| End dominant frequency |  | Dominant frequency measurement at the end of the signal (kHz). |
| dominant frequency slope | warbleR | slope of the change in dominant frequency through time ((enddom-startdom)/duration). Units are kHz/s. |

Table S2. Tukey-adjusted pairwise comparisons for the effect of treatment on individual-level and pair-level behavioral principal components that reached significance.

|  | **Treatment Contrast** | **Estimate** | **Std error** | **df** | **t-value** | **P** |
| --- | --- | --- | --- | --- | --- | --- |
| Individual-PC1 | Local-both - Local-plumage | 1.091 | 0.157 | 312.70 | 6.94 | **<0.0001** |
|  | Local-both - Local-song | -0.184 | 0.157 | 311.00 | -1.18 | 0.765 |
|  | Local-both - Foreign-both | -1.055 | 0.158 | 315.90 | -6.68 | **<0.0001** |
|  | Local-both - Control | -0.350 | 0.179 | 26.20 | -1.96 | 0.314 |
|  | Local-plumage - Local-song | 0.907 | 0.160 | 313.10 | 5.69 | **<0.0001** |
|  | Local-plumage - Foreign-both | 0.036 | 0.157 | 308.40 | 0.23 | 0.999 |
|  | Local-plumage - Control | 0.741 | 0.178 | 28.10 | 4.17 | **0.002** |
|  | Local-song - Foreign-both | -0.871 | 0.156 | 309.70 | -5.57 | **<0.0001** |
|  | Local-song - Control | -0.166 | 0.180 | 28.40 | -0.92 | 0.885 |
|  | Control - Foreign-both | 0.705 | 0.178 | 27.30 | 3.95 | **0.004** |
|  |  |  |  |  |  |  |
| Individual-PC3 | Local-both - Local-plumage | 0.356 | 0.123 | 312.10 | 2.89 | **0.033** |
|  | Local-both - Local-song | -0.308 | 0.123 | 311.90 | -2.51 | *0.091* |
|  | Local-both - Foreign-both | -0.317 | 0.124 | 315.60 | -2.57 | *0.079* |
|  | Local-both - Control | 0.082 | 0.166 | 22.50 | 0.49 | 0.987 |
|  | Local-plumage - Local-song | 0.048 | 0.125 | 313.50 | 0.38 | 0.996 |
|  | Local-plumage - Foreign-both | 0.039 | 0.123 | 308.50 | 0.32 | 0.998 |
|  | Local-plumage - Control | 0.438 | 0.165 | 23.20 | 2.65 | *0.093* |
|  | Local-song - Foreign-both | -0.009 | 0.122 | 307.90 | -0.07 | 1.000 |
|  | Local-song - Control | 0.390 | 0.167 | 23.20 | 2.34 | 0.168 |
|  | Control - Foreign-both | 0.399 | 0.166 | 22.80 | 2.41 | 0.150 |
|  |  |  |  |  |  |  |
| Pair-PC1 | Local-both - Local-plumage | 0.943 | 0.250 | 151.15 | 3.77 | **0.002** |
|  | Local-both - Local-song | 0.357 | 0.247 | 149.60 | 1.44 | 0.601 |
|  | Local-both - Foreign-both | 0.690 | 0.247 | 153.84 | 2.79 | **0.046** |
|  | Local-both - Control | 0.043 | 0.259 | 31.04 | 0.17 | 1.000 |
|  | Local-plumage - Local-song | -0.586 | 0.253 | 151.38 | 2.31 | 0.147 |
|  | Local-plumage - Foreign-both | -0.253 | 0.249 | 147.18 | 1.02 | 0.847 |
|  | Local-plumage - Control | -0.900 | 0.260 | 36.41 | 3.46 | **0.012** |
|  | Local-song - Foreign-both | 0.333 | 0.246 | 148.05 | 1.35 | 0.659 |
|  | Local-song - Control | 0.314 | 0.261 | 34.50 | 1.20 | 0.750 |
|  | Control - Foreign-both | 0.647 | 0.257 | 32.52 | 2.51 | 0.112 |

*‘Local-both’ refers to a mount presentation with a local plumage mount and female song; ‘local-plumage’ refers to a local plumage mount paired with a song from a population of the other plumage phenotype; ‘local-song’ refers to presentation of the local phenotype’s song paired with the other subspecies plumage; ‘Foreign-both’ refers to presentation of a color and song phenotype of the other subspecies; ‘control’ refers to emperor fairywren mounts paired with the local phenotype’s song.*

**Supplemental Literature Cited**

Araya-Salas, M., and G. Smith-Vidaurre. 2017. warbleR: an r package to streamline analysis of animal acoustic signals. Methods Ecol. Evol. 8:184–191.

Center for Conservation Bioacoustics. 2019. Raven Pro: Interactive sound analysis software (Version 1.6.1) [Computer software]. Ithaca, NY: The Cornell Lab of Ornithology. Available from http://ravensoundsoftware.com/.

Charif, R. A., A. M. Waack, L. Strickman, T. Krein, D. Hawthorne, A. Warde, D. Ponirakis, et al. 2003. Raven 742 software, the Raven 1.4 User’s Manual.
